# Developing a Framework for the Analysis of Program Notes Written for Contemporary Classical Music Concerts

**DOI:** 10.3389/fpsyg.2020.00376

**Published:** 2020-03-10

**Authors:** Diana Blom, Dawn Bennett, Ian Stevenson

**Affiliations:** ^1^Music, School of Humanities and Communication Arts, Western Sydney University, Penrith, NSW, Australia; ^2^School of Education, Curtin University, Perth, WA, Australia; ^3^Faculty of Arts and Social Sciences, University of Technology Sydney, Ultimo, NSW, Australia

**Keywords:** program notes, audience, music education, concerts, classical music, western art music, performance research, music composition

## Abstract

At classical music concerts, a program note is the usual medium for communicating information about the music to be heard and performed. Although there may be crossover of information, the program note is distinct from the CD cover note, from notes contained within a musical score note, and from a composer’s directions for performers. With a focus on contemporary classical works in the Australian context, the researchers’ aim in this study was to develop and test an analytical frame of informational categories with which to examine program note content. Three extant studies – one scientific, one phenomenological and one semiotic – informed the development of an initial theoretical framework for program note analysis. This was tested through the analysis of program notes (*n* = 30) from each of three writer cohorts: composers, professional writers, and higher education students. The analytical frame revealed different emphases of information categories among the three program note writer groups, with a more sophisticated combination of categories used by the professional writers and composers. This has implications for the teaching of program note writing in tertiary performance institutions, encouraging diversity of student content without extinguishing personal insights.

## Introduction

At classical music concerts, a program note is the usual medium for communicating information about the music to be heard and performed. Despite this, there is almost no research on the role and impact of program notes; neither is there research on program note content or structure. With a focus on contemporary classical works in the Australian context, the study reported in this article aimed firstly to develop an analytical frame with informational categories with which to examine program note content; and secondly to trial the preliminary frame by analysing program note content written by students, composers and professional writers in relation to the informational categories, grammatical person, word count and program intent.

### Review of Literature

Despite research that emphasizes the importance of the engaging with music audiences, there exists limited scholarly writing about the program note. And yet audience engagement and commitment is a long-held concern for live music scholars and practitioners (Pitts, 2005). One of the complexities of audience research is that audiences cannot be treated as a homogenous group. Sloboda and Ford (2012), for example, emphasize that audience engagement strategies might need to differ for younger audiences. Dobson, 2010, p. 111) writes that “feelings of inclusion and participation in the performances” are important predictors of enjoyment among concert-goers who are new or inexperienced. Burland and Pitts (2014) add that there is a strong temporal dimension to the experience of viewing an ephemeral event such as a music concert: their cyclical process of being an audience members is reminiscent of Feld’s (1994) experiential anchors in its reference to audience members’ accrual of lived personal and social experiences of music performance. Post-performance engagement forms part of the cyclical process and might involve both informal discussions and expert reviews (Alessandri et al., 2011; Dobson and Sloboda, 2014). These acts of responding, reflecting, re-experiencing and actively participating in music are informed by multiple informational, experiential and socio-emotional stimuli. Chief among these informational stimuli in the live music context is the written program note, which is the most common medium for communicating information about music yet to be heard and performed.

The first of only three studies to analyze the impact of program notes on listeners was conducted by Margulis, 2010, p. 298), who found that listeners who have read a program note “are more likely to listen [to the music] in terms of the concepts just encountered” rather than to let the music simply “wash over” them. Margulis (p. 298) observed that “arts presenters who rely on program notes assume the conceptualization they offer increases pleasure,” but her study concluded that this is not necessarily the case.

Following on from Margulis’ work, Bennett and Ginsborg (2018) gave two performances of unfamiliar music to the same audience and explored audience reactions by sharing the program notes only after the first performance. All listeners responded differently to the music once they had been given the program notes. In line with Margulis, only 39% of listeners reported that the program notes had had a positive impact on their listening experience. Of interest, more experienced listeners in Bennett and Ginsborg’s study were far more likely to reject the program note information in favor of their own interpretation, particularly if they had experiences of music making; this suggests that the impact of program notes is influenced by the musical experience and knowledge of listeners.

Bennett and Ginsborg (2018) suggest that the impact of program notes on listeners might relate to familiarity with the music being performed. However, they warn that the relationship between familiarity and liking is not yet understood. North and Hargreaves (1995), for example, found a positive association between familiarity and liking, whereas Brown and Knox (2016) concluded that audiences seek novelty rather than familiarity, and Thompson (2007) found no relationship between familiarity and audience members’ enjoyment of a classical concert.

In the third of three extant studies on the music program note, Blom et al. (2016) investigated the purpose and role of a program note written by contemporary classical music composers about their own music. The study identified five key themes which we describe in turn.

The first theme relates to the intended purpose of a program note, which can be to guide or to direct the listener. As such, program notes typically provide the titles and composers’ details for works within the concert program alongside information on poetics or aesthetic suggestions of the piece, the personal experience of the composer, and links with other music works or art works of other arts disciplines that informed the piece. A program note can also include performer notes which might be of interest to listeners: for example, an alternative tuning or tonguing technique.

Blom, Bennett and Stevenson observe that a program note could be considered a vital inclusion for performances of abstract music, however, in line with Margulis’ observation they note that a program note for abstract music might mislead audiences by imposing an external framework. In general, an argument might be made that musical works shouldn’t require a program note to be effective and that reductive listening allows a focus on the sound itself, removed from anecdotal, referential preconceptions of the piece to allow listening which is free from prejudice. A program note might also present a work as overly complex or intellectual.

The second theme is that the program note can seek to create “ideal listeners” by preventing modalities not in accord with the composer’s intentions. The program note can also guide listeners whilst not limiting interpretation or the listening experience; by retaining some ambiguity, it can inform and contextualize but not essentialize what the listener hears in the music. A program note can therefore provide a subtle track, leaving some mystery in the air and suggesting rather than imposing a framework for listener engagement.

The third theme identified by Blom, Bennett and Stevenson is that a program note can help to shape the performer’s interpretation of a work. This might take the form of programmatic notes or descriptions of the sounds and timbres the composer would like to evoke.

Fourth, a program note can inspire the listener to become absorbed in listening to the music, thereby adding a dimension which enables the listener to become more engaged or involved in the music. This can be a way to access the work, promote understanding, and reveal what the piece is trying to explore and achieve.

The fifth and final theme is that the program note can be a collaborative tool between composer, performer and listener by sharing larger artistic concerns, giving additional insights and helping both performers and listeners navigate the piece. This generates dialogue between performer, listener and composer, communicates the composer’s intention through the performer to the listener, and encourages the listener to empathize with the composer’s intentions. Several roles for a program note emerge from the literature: restraining the listener, guiding the listener, scaffolding familiarity versus novelty in the repertoire, helping shape the performer’s interpretation, inspiring musical absorption, and acting as a collaborative tool.

### Program Note Content

For Wingell (2009), the program note informs the audience what there might be to listen for. Similarly, Harries, 2017, p. 80) finds that program notes might “guide listeners toward interpretive responses.” Taking a broader view, Scaife, 2001, p. 4) advises performance students that program notes should be “pertinent and persuasively written, with thoroughly researched and well-balanced commentary.” Wingell (2009) adds that composer input to a program note helps avoid the danger of the audience judging a recent work against the wrong set of expectations.

Professional program note writer Leonard Burkat (1985, p. 2) agrees that the input of the composer can be invaluable. Burkat suggests that when composers are asked what they would like to be written about their piece, they can sometimes “express thoughts and feelings in words so precisely and effectively that no one else’s will do….” Irvine (1999) and Holoman (2008, p. 84) add that program notes need to be relatively short as there is usually little time for reading them. As such, the writer should consider different styles of program note such as the “a sidebar with a few points to listen for during the performance.”

Scaife’s “well-balanced commentary” can contain three types of content information. The first of these is contextual information, which can inform listeners about of the personal experience of the composer or about other musical works or works of art that informed the piece (Blom et al., 2016). The commentary might also state where the work fits within the evolution of musical styles (Wingell, 2009). Background information (Irvine, 1999) such as details about the first performance, publication date (Holoman, 2008) and special circumstances surrounding the composition (Holoman, 2008; Wingell, 2009) might be included, as might information about the performer (Blom et al., 2016).

The second type of information relates to descriptive and expressive aspects of the music. This information might relate to the poetics and aesthetics of the piece (Blom et al., 2016) or to the “content” of the music (Scaife, 2001). Music critic Turner (1933, in Scaife, 2001, p. 7), however, warns of descriptive notes which are “useless to everybody, and positively harmful to those who are seriously trying to understand the art of music” because they are “remote from the useful function of the annotator and critic.” Turner gives as an example writers who describe symphonies “as if they were sunsets or battles, or election conflicts between the good and evil parties in the Universe” (ibid).

The third type of information in a program note relates to technical details such as compositional structure and devices. However, most of the advice to program note writers warns against using “technical analysis that will make no sense to the majority of the audience” (Wingell, 2009, p. 106) on the basis that this will lessen communication with a majority of the listening audience (Scaife, 2001).

At the heart of a program note is analysis of the music. For composer and theorist Dubiel (1999/2001), p. 274),

… analyses of music are more likely to be valuable as consciousness-raising exercises – or as the tools for such exercises – than as renderings of the content of musical experiences. What they might help to raise consciousness about is what goes into a hearing, what there might be to listen for, that no one had thought to listen for (perhaps even while listening for it).

Dubiel’s account is a fair description of the role of the program note as a consciousness-raising exercise for the listening audience.

Harries’ study into “attitudes toward program notes, and their influences on responses to music” (2017, p. iv) asked two participant groups, musicians and music novices, to rank the most and least interesting aspects using a list of five program note features. The musicians ranked historical background of music highest, followed by narrative details of music, composers’ backgrounds, compositional/structural analysis, and performers’ biographies. For the novices, narrative details of music was ranked first, then historical background of music, composers’ backgrounds, performers’ biographies and compositional/structural analysis. All participants believed that program notes “played a role in facilitating musical experience … [assisted] with directing attention and focus, provided insight into appropriate listener responses, or helped assess the quality or accuracy of a performance” (2017, p. 43). All participants also wished to be provided with a program when attending concerts; however, they expressed a range of views on the *amount* of interpretive guidance they preferred.

Harries’ study suggests that musicians are likely to prefer to form their own interpretative responses to music, whereas novice listeners welcome interpretative guidance; this finding mirrors that of Bennett and Ginsborg (2018). Most of Harries’ participants appreciated “guidance toward composers’ or performers’ intent, or … important narrative or imagery background” (p. 72); narrative programs in particular were found to have a positive effect on participants’ understanding of musical excerpts. Musician participants were interested in the training and professional background of the performers, while novices liked to read about performers’ musical tastes and the “motivations for a performer’s repertoire choices” (p. 46). Musicians were more interested in compositional analysis than were novices. Harries concluded that the ability of programs to create a connection between performers, composers and the music itself, offers musicians another way “to provide satisfying musical encounters” (p. 44).

Arguing the need for a “retooling” of technical descriptions of music within music analysis, Lochhead, 1998, p. 3) outlines the need to broaden analytical information. Her commentary stems from criticism by three major 20th century musicologists (Kerman, Trietler and McClary) relating to “technical descriptions” and technique-based analysis of music. Lochhead (p. 3) explains that the musicologists found these approaches problematic because

… they (1) conceal the conceptual and ideological underpinnings of their accounts of what music does, (2) take an exclusionary approach to musical explanation, eliminating the historical and critical context that surrounds understanding, and (3) ignore the expressive, or “signifying,” features of musical meaning.

Here, Lochhead is emphasizing the value of contextual information in providing access to a deeper appreciation of the music by a broad range of listeners. She also encourages a clearer engagement with the meanings linked to musical content which has been obscured by a focus on the techniques of composition. Lochhead (p. 6) recognizes the “current interest in music as experienced by listeners, listening-oriented criticism and explanation” as part of a broadening of music analysis, and she argues that “this ‘variant understanding of a world of music’ requires a retooling of the technique in order to reveal ‘precisely’ and ‘productively’ this dimension of the musical phenomenon.” She also reminds us (p. 5) hat

… a technique-based account of music will likely not have a revelatory function for such listeners [to whom] … technical accounts of music at either a low- or high-level of explanation may seem like a “foreign language” … because they have no practical engagement with the technique.

This is precisely the same view communicated by several writes about program note content, including that of professional program note writer Leonard Burkat, 1985, p. 2) who found that some composers, when writing about their own music, “…cannot think of anything to tell except technicalities that will mystify the lay listener and distract attention from the music itself.” Yet, as noted above, Harries’ (2017) musician participants preferred the inclusion of technical compositional information in their program notes.

Our review of scholarly and gray literature revealed one further type of information, found in an program note written for the New York Philharmonic Society, 1846. In this program note, the professional program note writer gives a candid “personal value” opinion of the *War Jubilee Overture* by Lindpaintner, writing: “tho’ not possessing any great depth as a musical composition, it is remarkable for the vigor and power of the instrumentation” (archives.nyphil.org). Such personal value comments can be categorized as descriptive comments.

Of the two examples of “good” program notes given by Scaife (2001), the program note for the most recent of the works, the *Sonata in E flat minor* (1905) by Janáček, includes three types of information. There is historical contextual information in the first paragraph. The rest of the note comprises largely technical analysis with expressive descriptive adjectives and references to the poem Janacek wrote about a tragic event which formed the impetus for the sonata. Scaife’s program note example says (p. 13): “The second subject provides reflective calm in an otherwise tempestuous movement. It is likely that the composer was recalling the crowd scenes and events of the poem while writing this highly-charged music, which contains much anger and frustration.”

In summary, program notes are texts. As such, they can be viewed as “the product of interpretation and claim in social life and should be seen both as motivated (i.e., produced for purposes), and as accounts (i.e., versions not in some referential way)” (Stanley, 2013, p. 5). Stanley is referring broadly to texts which are documents of life, including letters, but her thinking is relevant for program notes as they are products of interpretation and claim, produced for a purpose, and created as written accounts of a piece of music. All this is contained within contextual, descriptive and technical information. In writing for (not about) theatre performance, Harris and Holman Jones, 2016, p. 1) find that “writing and performance are too often contrasted as different and at times contradictory practices,” with the writing being a record of the event and the performance being embodied. Harris and Holman Jones, 2016, p. 1) assert that “writing and performance are two arms on the same body. If performance-making is a practice of inscription, writing is equally a physical practice. It is a making practice, a creative practice, and critical practice….” A program note plays a different role in a music performance, intended, primarily, to guide the audience rather than the performer, yet it is a creative and critical practice of making which can engage or disengage a listener.

## Materials And Methods

The study was conducted in two phases. We began in phase 1 with a review of scholarly and gray literature. This led to the theoretical framework shown at Table 2. The framework was then tested, in phase 2, for adequacy as a tool for descriptive content analysis. Phase 2 involved the analysis of ten program notes from each of three writer cohorts (30 program notes in total): composers, professional writers, and higher education students (see Table A1 for details).

For consistency, the program notes were focused on the Australian context: that is, program notes written about Australian contemporary classical works by professional program note writers, Australian composers, and higher education students. The programs written by professional writers and Australian composers were selected from programs of concerts given over the previous 10 years; each program featured a different musical work. The student program notes were drawn from students at a single higher education institution and were written over the previous 20 years period. The longer time frame was required as students are not required to perform Australian repertoire; hence, there were fewer examples.

Drawing on aspects of Dampier’s (2013) methodological practices when analyzing historical letters, the three researchers, all active both as musicians and music academics, each independently made a close reading and re-reading of the program notes, situating the program note content in relation to the analysis framework. In order to evaluate the descriptive adequacy (Ashworth, 2000) of the analysis categories, sections of the entire text of each program note were allocated to one of the five codes.

Generally, analysis categories aligned to text of between one and ten sentences. In nearly all cases, codes were applied at sentence boundaries. Where compound-categories were identified, single categories were replaced with compound codes. Following initial coding of the 30 program notes, the three sets of analysis were tabulated and compared. Where differences were observed, detailed discussion led to consensus. During this final stage, minor adjustments were also made to the application of codes until all three researchers were satisfied that the existing code definitions had been applied consistently.

Subsequent to coding the program notes, the following information was noted: the percentage coverage of each category of content information; grammatical person (whether first or third person writing was used); total word count for each program note; and categories or styles outside the expected. These were defined as cases “likely to upset your thinking” (Becker, 1998, p. 87), or which might challenge the adequacy of analysis categories.

Coding was applied using the text formatting and word count features of Microsoft Word. Word count percentages, averages and standard deviations (STDEVP) were calculated using Microsoft Excel.

To protect their anonymity, students program writers are identified as Student A to Student J and student composers are given pseudonyms. The notes written by professional writers and composers are in the public domain and these writers are identified in each case. For all writers, we include the year in which each note was written.

## Findings

### Phase 1: Developing a Theoretical Framework

From the literature review, three studies in particular – one scientific, one phenomenological and one semiotic – laid the foundation of a theoretical framework of informational categories with which to analyze program note content. This combination of studies suits analysis of a document containing musical and personal information – the program note. In her study of the effect of two different types of music description (or no description) on 16 people without formal musical training when listening to excerpts from Beethoven String Quartets, Margulis (2010) differentiated between affective, imaginative language (dramatic features) and objective, structural language (structural features). The study found that participants preferred excerpts preceded by no description: short text descriptions (program notes), whether dramatic or structural in content, reducing enjoyment for the listeners. Margulis (p. 295) posited that “listeners may seek to be swept away by the music” and that conceptual listening or listening to music in terms of linguistic descriptions may be less enjoyable. Margulis also noted that listeners new to contemporary music may benefit from a descriptive note, although the results of Bennett and Ginsborg (2018)’s study, discussed earlier, cast doubts on this assumption, particularly in the case of more experienced listeners.

Ferrara’s (1984) framework for a listener’s phenomenological analysis when listening to contemporary classical works was derived by analysing listeners’ observations when hearing Edgard Varčse *Počme Électronique* five times. Ferrara concluded that the first stage of listening is a syntactical orientation in which people listen openly to “everything.” Listeners then begin to separate sounds for their individual and phonemic qualities before considering the meanings behind, or relationships between the sounds: the semantic meaning of the music. Next, listeners consider ontological meanings: the work in its entirety. This might include, for example, the historical life-world of the composer and the circumstances in which the work was written.

In terms of five repeated hearings, Ferrara summarizes the process as follows:

**Table:** 

Time 1	Everything	Open listening
Time 2	Sounds and their phonemic qualities	Syntactical meaning
Time 3	Meanings behind/relationships between sounds	Semantic meaning
Time 4	Relationships beyond the music	Ontological meaning
Time 5	Everything	Open listening

Mirroring Burland and Pitts (2014)’s emphasis on temporality, Ferrara was careful to point out that the ontological world of a composer’s “lived time” – the time and context in which a composition was created – is unique, and that the outlooks and values that informed this lived time are impermanent. Ferrara also considered the role of feeling, movement, gesture, space, temporal relations and tactile qualities.

Margulis (2010) identified affective, imaginative language (dramatic features) and objective, structural language (structural features). In Ferrara’s study, listeners often used what Margulis refers to as dramatic text to describe the sounds (syntax) and the sound meanings (semantics), and also to describe ontological references. The latter might be literal (the metallic sounds of a clock ticking) or metaphorical (eerie, like a still ocean). Margulis’ structural text refers to structural features such as modulations, instrumentation and pitch. Combining the two, the following initial theoretical framework emerges (see Table 1).

**TABLE 1:** Initial theoretical framework combining Ferrara (1984) with Margulis (2010).

Margulis	Ferrara	Explanation	Focus
Structural features	Syntactical meaning	Sounds are considered in relation to their individual and connecting phonemic qualities. Semantic and ontological meanings are ignored.	Sound
Dramatic features	Semantic meaning	Interpretation of sounds and sound relationship at a fundamental level: relationships to known origins and complex understandings of the sounds *within* a work.	Possible references
	Ontological meaning	Interpretation of a whole work, which may include historical and socio-political contexts *beyond* a work.	Relation to life-world

Although Margulis did not consider ontological or poietic language in her study, three basic categories are noted in the literature: technical (syntactical); descriptive (semantic); and contextual (ontological); however, we contend that two further compound-categories emerge. In practice, categories 1 and 2 are often combined in program notes such that essentially technical descriptions lead to expressive assertions. For example, in Timothy Munro’s (2016b) note for composer Alexander Garsden’s *We Never Come Here*, Munro observes that “maniacal, irregular tapping puts teeth on edge. Slow pulsing summons a wall of sound. Each jump-cut is hard, unexpected, communicating uncertainty, agitation.” Here are three short sentences, each beginning with technical information and moving immediately to descriptive assertions. This can be thought of as category 4. Similarly, essentially expressive assertions are often supported by technical features. Rachel Campbell’s note for composer Michael Smetanin’s *Swell* (2008) links the work’s central structural concept with its title. This can be thought of as category 5.

The notion of a wavelike swelling motion … underlies … aspects of the work: certain wavelike gestures, and the way the clarinets expand out from a restricted, close set of pitches at the opening to a larger musical space as the first section progresses. Further expansion and support is then received from the gradual entries of the other instruments.

In his book on semiological analysis, Nattiez and Abbate (1990) develops a tripartite model for musical analysis. The three dimensions of the symbolic phenomenon of music (poietic, neutral/trace, esthesic) suggest three processes which are the “objects of analysis” (poietic processes, the material reality of the work-sound/score/performance, esthesic processes). Nattiez offers three corresponding families of analysis:

1. The poietic dimension: aspects of the process of creation, deliberations (by the composer) on what must be done to produce the work, operations on materials, the production of the work;
2. The neutral/trace dimension: an “objective” description of the properties of the work, its form/structure, etc.; and
3. The esthesic dimension: assigning or constructing meaning from the work in the course of active perception.

We also suggest that for simplicity of analysis, the contextual (ontological) and poietic categories, though different in emphasis, might be combined to refer to the imaginative, biographical, historical, contextual, practical and productive aspects of the composer’s work. The “personal value” comments of the anonymous program note writer of 1844 align with category 2 (descriptive). The thinking of Ferrara, Nattiez, Margulis, and other commentators discussed earlier in the paper, lead us the refined theoretical framework presented at Table 2.

**TABLE 2:** Analytical framework for program notes in music.

Category	Source analysis				Focus
	Ferrara	Nattiez	Margulis	Other writers	
Technical	Syntactical	Neutral	Structural	*Technical* Turner, 1933; Burkat, 1985; Lochhead, 1998; Wingell, 2009; Harries, 2017	Sound, musical structures, features, form
Descriptive	Semantic	Esthesic	Dramatic	*Expressive meanings* Lochhead, 1998	Possible references,
				*Poetics and aesthetics* Blom et al., 2016	meanings, interpretation
				*Content of music* Scaife, 2001	
				*Personal value* 1844 writer	
				*Narrative details* Harries, 2017	
Contextual	Ontological	Poietic	N/A	*Historical and critical contexts* Lochhead, 1998; Wingell, 2009; Blom et al., 2016; Harries, 2017	Relation to life-world, historical or production creation
				*Dramatic* Turner, 1933	
				*Personal experience of composer* Holoman, 2008; Wingell, 2009; Blom et al., 2016; Harries, 2017	
				*Personal details of the performer* Harries, 2017	
				*Background information* Irvine, 1999	
				*First performance, publication date* Holoman, 2008	
Technical-Descriptive	Syntactical-Semantic	Neutral-Esthesic	Dramatic		Essentially technical leading to descriptive assertions
Descriptive-Technical	Semantic-Syntactical	Esthesic-Neutral	Dramatic		Essentially descriptive supported by technical evidence

### Phase 2: Testing the Framework With Program Note Content

As the standard deviation of population figures at Table 3 show, the percentage-of-word-count distributions for the three groups of writers were not simple uni-modal distributions; however, the averages indicate the prevalence of each content category. The most prevalent category of program note content from all three writing cohorts was *contextual* information: information which details influences from an historical context, the arts, aspects of the production or creation process, and/or relations to the life-world of the composer.

**TABLE 3:** Content of program notes written by professional writers, composers and students.

	Average percentage and std. dev. (population) of content type				
Writing cohort	Contextual	Descriptive	Technical	Descriptive-technical	Technical-descriptive
Professional writer	36.95%	10.52%*	26.66%	8.24%	17.69%
	SD 22.73%	SD 11.88%	SD 25.58%	SD 16.96%	SD 11.29%
Composer	33.89%**	32.86%	15.16%	1.50%	14.45%
	SD 32.26%	SD 41.41%	SD 28.47%	SD 4.50%	SD 18.71%
Student	52.50%	12.20%*	17.40%	0%	13.40%
	SD 26.03%	SD 20.59%	SD 27.37%	SD 0.00%	SD 19.33%

*Includes personal value (Professional- 1.62%, Student- 4.5%). **Includes contextual-descriptive (Professional- 2.98%, Composer- 2.17%).

Professional writers’ program notes favored *contextual* information (37%) with *technical* the second most used content style (27%). *Technical-descriptive* information was used for 18% of the total word count across all program notes; however, information leading with expressive content (*descriptive* and *descriptive-technical*) was used least, at 11% and 8% respectively).

*Contextual* information provided by professional writers for contemporary Australian works included references to the commissioning process often for specific performers or ensembles, or the venue in which the program was presented. For example, Timothy Munro’s description of Natasha Anderson’s *Nowhere and Forever* (2016a) introduced the café featured in a television drama which inspired the work, and then linked this to the venue in which sounds for the work were collected. Munro wrote:

… like the cafe, Carriageworks [a Sydney performance venue with a rich railway yard history] itself is caught between past and present, a building haunted by memories, yet transformed for a new generation, an anachronism.

For older works, links were made to the composer’s *oeuvre*: for example, Michael Hooper, writing about David Lumsdaine’s *A Tree Telling of Orpheus* (1990), noted that

many of Lumsdaine’s works begin simply. The orchestra piece *Hagoromo* begins with the growth from a single pitch to changing harmony, a very similar gesture to that which begins *A Tree Telling of Orpheus*.

The professional writers’ use of *technical* content described specific musical features: for example,

The melodic material is conceived as simple contours (a sequence of scale degrees) rather than specific pitches. (Program note by Ricketson, 2012);

*Aria for Edward John Eyre* – an hour-long *tour de force* for soprano, ensemble and electronics – also begins with a single pitch, then a chord, and then a melody. (Program note by Hooper, 2014);

The supporting ensemble is a mellow and flexible combination of three woodwinds (with emphasis on low doublings – alto flute, cor anglais, bass clarinet) and string trio, with the addition of a guitar to suggest Orpheus’ lyre (program note by Gyger, 2014).

Composers’ program notes favored *contextual* (36%) and *descriptive* (33%) information with *technical* information making up 15% and *Technical-Descriptive* representing 14%. In line with the professional writers, some of the composers’ program notes included *contextual* information about the ensemble itself, its aims, ethos, aesthetic, which begins the individual notes for pieces.

*Descriptive* information was used by composers to describe their musical intent and outcomes. Some composers included *contextual* comments about intimate events related to the writing of the piece. Clare Maclean captures the *context* of her work for viola and piano in two sentences.

“In the sea paths” is a phrase from the Anglo-Saxon poem, the Seafarer. The poem’s imagery of sea paths as paths of life resonates with symbolism of the sea as the soul or the subconscious in literature and mythology.

None of the content represented “personal value” comments, although composers often talked of their experiences when writing about their own work. The category of *descriptive* content is exemplified by composer Mary Finsterer writing about her piece *Biographica* (Composer note, 2017).

The composition aims to catch moments in the journey and demise of an eccentric, wondrous soul from the beginnings of our modern age. It’s like a visit to a great portrait gallery, full of different paintings – but all depicting the same person.

Student program note writers used a narrower range of content types. *Contextual* content was the most prevalent at 53%, with *technical* (17%) and *descriptive* (17%) used less often. Some student program notes included a “personal value” comment about their composition or their performing concerns; these comments were only seen within the student writing. For example, student performer Student A reflects concerns about the difficulty of the performance: “I will aim to play what [the composer] has heard and written to the fullest degree.” This type of content was not found in the composer and professional writers’ notes as it is likely to create a strong effect on the reader. However, the comments do reflect the afore-mentioned 1844 program note writer who gave an authoritative, candid, negative “personal value” comment about a composer’s work.

Within the student writing, some use was made of two content styles within a single sentence group; this was always *technical-descriptive* (13%) and not *descriptive-technical* content. The following example begins with *technical* detail that links to evocative *descriptive* content:

The piece uses Western instrumentation to mimic a more traditional Japanese ensemble sound, consisting of sporadic percussion in free time that symbolize the first flickers of ember in the flame. (Program note by student D)

Professional writers and composers often used sentence groups with compound-categories, in particular *technical-descriptive* material with some use of the *descriptive-technical*, depending on which type of information was privileged within the sentence. In the following example, terms such as “absurd beatboxer” and “ubiquitous symbol” pushed *technical* material into the *descriptive-technical* category:

Composed especially for the Co-Artistic Director, Hi Hat and Me reduces the drum kit – the ubiquitous symbol of pop music’s beat – to a mere hi hat. With such limited resources on which to stake their virtuoso coordination, the percussionist instead turns to vocalizing like an absurd beatboxer. (Professional program note by Ricketson, 2012)

Some sentence groups extended our categories to consider *descriptive-contextual* material: for example,

Anderson’s works are unsettling sonic mystery-boxes. This Australian composer, musician and installation artist aims to create “idiosyncratic” sounds that explore intense experiences, the abject and the uncanny. (Composer program note by Munro, 2016a)

Here, the *contextual* information concerns the composer. It is written by someone other than the composer and yet seeks to represent the composer’s intentions in relation to expressive responses to the sounds.

In summary, shown at Table 3, professional writers had the highest percentage of *technical* information in their program note content, composers had the highest use of *descriptive information*, and student program notes had the highest percentage of *contextual information*.

#### Word Count, Person, and Program Intent

Other aspects of program note content considered in the study were word count, use of first and third person and program note intent.

Program note word count was higher for professional writers than for program notes written by composers and students (see Table 4).

**TABLE 4:** Program note content (*n* = 30).

					Characteristics of the program note (count)			
Writer	1st person	3rd person	Word count (average)	Min/Max	Content		Intent	
Professional writer	2	8	302	203/517	Contextual	5	Guide or direct listeners	10
					Descriptive	5	AND encourage interpretation	4
					Historical	2	Provide historical context	1
					Interpretative	7	Link work to its presentation within the venue	1
Composer	3	7	105	32/204	Historical	2	Provide history	1
					Context		Provide context	3
					Draw listeners in	6	Context/instruments	1
					Direct listeners	1	Guide listeners	5
Student	6	4	172	70/406	Influences	7	Guide or direct listeners	8
					Personal challenge	1	Collaborative tool	2
					Theoretical frame	1	Both the above	2
					Compositional thinking	1		

Analysis of grammatical person revealed that third person was used in the majority of the writing by composer and professional writers and first person was used by the majority of student writers. In one case, the professional program note writer was also a member of the performing ensemble; in this case, the writer used first person to describe his relationship with the composer and his music, and third person to describe the music itself. In three cases, composers used first person to provide *contextual* information about the genesis of the work. In contrast, student composers and performers were more inclined to write about their own experience of developing the work for performance and their influences. This first person presentation style helps to account for the higher proportion of contextual information in student program notes.

To examine intent, we drew on three of Blom et al.’s (2016, p. 9) five key themes of program note intent: “to guide or direct the listener or performer; to shape the performer’s interpretation; and as a tool for collaboration between the composer, performer, and listener.”

In professional writers’ program notes, *contextual* information provided historical context, sometimes linking the work to its presentation within the venue as in the *Carriageworks* example given earlier. *Descriptiv*e information encouraged interpretation and sought to guide or direct the listener.

In the composers’ program notes, *contextual* historical information, information about instruments, and *descriptive* information about influences on the work, were written with the intent of guiding and directing the listener, and drawing the listener into the music. Student program note writers gave *contextual* information on influences, *descriptive* information on personal challenges and *technical* information on compositional thinking, all with the intent of guiding and directing the listener. Both the composers and students designed the program note as a collaborative tool between the writer and the listener. Student performers sometimes made overt connections between the composer, the performer (themselves) and the listener: for example, “I haven’t had much experience with performing prepared piano at all, henceforth this piece being a challenge that I hope to interpret it supremely” (program note by Student A).

No “personal value” comments regarding a writer’s like or dislike of a piece were encountered; however, both student and established composers used first person language in their *descriptive* writing about their aesthetic reasoning. For example, composer Mary Finsterer (2017) described her creative objectives thus: “In Biographica I’ve focused on capturing an essence of Renaissance music by filtering it through a contemporary lens”; and student H wrote, “This indeed is a challenge to perform and I would especially like to thank [the composer] for the hours he has spent helping me prepare for this performance.”

## Discussion and Conclusion

The new analytical frame provided a useful guide for the investigation of program note content. In terms of content coverage, the framework categories were generally shown to provide descriptive adequacy. Although *technical-descriptive* and *descriptive-technical* compound-categories were identified at Table 2, our use of the frame suggests the addition of a *descriptive-contextual* category. This would have accounted for around 3% of the content of professional writers and 2% of the composer’s content. This type of writing, whereby two content styles are combined, might temper the potential for *technical information* alone to become dry and alienating (Scaife, 2001; Wingell, 2009), and in doing so may heighten the usefulness and enjoyment of the program note text for both reader and listener.

Program note informational content included a range of categories, the role of which was to guide and direct listeners through information which is variously technical, descriptive and/or contextual. The program note is not a benign piece of writing; rather, it is a documentation or narrative of a musical work and the life from which it came (Stanley, 2013).

In this study, the three groups of program note writers adopted different content styles for their program notes and brought different perspectives to that writing. This perspective difference is perhaps of particular importance when the work is contemporary and the composer’s voice can be heard without being mediated through another writer. The distribution of content types within a single program note varied across the three cohorts of writers, but all three cohorts favored *contextual* information over the other content types.

Professional writers and composers were able to combine content styles in one or two sentences. Professional program note writers also included a higher proportion of *contextual* and *technical* content than composers and student writers to guide and direct the listener. These content types are to be expected when the writer is not the composer or performer with inside knowledge of the work and is therefore writing for a concert audience with which there will be no direct engagement.

*Descriptive* writing about a musical work received the highest use by composers. This aligns with Burkat’s (1985) assertion that a *descriptive* style of information comes best from the composer. Although student program note writers used a narrower range of content styles in their program notes, they often offered a frank and personal view of their compositional or performance concerns. These personal views were refreshing to read, although the communication of specific worries and concerns could negatively impact the enjoyment of the listener.

In the higher education performance environment, the program note can be an authentic assessment task for performance and composition students, enabling the development of professionally oriented writing skills and requiring deep thinking about repertoire, context, influences and compositional process. Some institutions offer advice on how to write these notes, yet the instructional texts largely fail to communicate that program notes are far from neutral documents and that their purpose and structure varies. The findings of this study offer an opportunity for students to learn how to shape program note content aimed to engage the audience in a variety of contexts, through a marriage of descriptive, technical and contextual information. This is a skill which can be taught, learned, and refined over time.

Writing from the context of contemporary interest in the listener experience of music, Lochhead (1998) argues for a “retooling” of the technical descriptions of music in order to avoid dry descriptions which alienate listeners through overly complex or technical language. Dubiel (1999/2001) writes that analyses of music – a focus on the technical aspects of music – could be seen as a consciousness-raising exercise. It follows that finding a way to effectively communicate some of this technical information to listeners might enhance their enjoyment and understanding of a musical work, although Harries (2017) found that the listener with musical training welcomes such information more than the musical novice. The three program note writer groups all worked closely with technical information in their musical lives and they would have understood technical information relating to the work. This study suggests that writers could organize their writing in order to communicate technical information through a combination of technical and descriptive (and perhaps contextual) information in various combinations; this might be a way of retooling the technical descriptions.

Although the study did not examine different styles of program note format, such as a sidebar with key listening points (Holoman, 2008), we noted the use of personal text such as relationship to self, effect on self, emotion, and the use of metaphor. And two of Ferrara’s, 1984, p. 9) extra qualities (feeling, movement, gesture, space, temporal relations and tactile) were identified: namely, feeling and tactile qualities in Campbell’s gendered description – “The inspirations behind the Beauty Boxes in this work were the make-up and jewelery boxes of past eras, objects made up of fascinating compartments containing materials to enhance feminine presentation” (Campbell, 2008).

Professional program note writers wrote the longest program notes, although we note that length is usually determined by a publisher or, in the case of students, a teacher; there is also little time for reading program notes (Irvine, 1999; Holoman, 2008), so it is unsurprising that all program notes were relatively short.

Finally, the findings suggest that writing a program note is always a collaborative relationship, a point raised by Harries (2017) and Blom et al.’s (2016) studies. The program note is a collaborative tool between program note writer/composer and the listener, sharing larger artistic issues and enabling additional insights. For the student program note writer, the program note often outlines the collaborative relationship between performer/program note writer, composer and listener with concerns and rehearsal process discussed. This generates dialogue between performer, listener and composer, communicates the composer’s intention through the performer to the listener, and encourages the listener to empathize with the composer’s intentions. It has the potential to give confidence to the audience in their exploration, interpretation and enjoyment of the music, positioning listeners as active participants who make a creative contribution through their personal interpretation. There is also room in this collaborative relationship for the concert programmer and producer. And from the temporal perspective, there is room for post-listening experiences ranging from post-concert conversations through to expert commentaries such as critical reviews (Alessandri et al., 2015). This communication is therefore multi-directional, with the program note activating the listener’s participation.

## Data Availability Statement

The datasets generated for this study are available on request to the first author.

## Author Contributions

All authors made substantial contributions to the conception or design of the work and the acquisition, analysis, and interpretation of data for the work; drafted the work and revised it critically for important intellectual content; approved the final version to be published; and agreed to be accountable for all aspects of the work in ensuring that questions related to the accuracy or integrity of any part of the work are appropriately investigated and resolved.

## Conflict of Interest

The authors declare that the research was conducted in the absence of any commercial or financial relationships that could be construed as a potential conflict of interest.

## Appendix

**TABLE A1:** Program notes cited in the article, by writing cohort.

Author	Year	Work	Source
**Program Notes Written By Composers**			
Diana Blom	2011	*After the rain*, for four pianos	Audio-visual collaboration with artist/film maker Noeline Lucas, first exhibited at the Bathurst Regional Gallery, January 2011.
Mary Finsterer	2017	*Biographica*, chamber opera	Sydney Festival concert program, City Recital Hall, 19 January 2017, Sydney Chamber Opera in association with Ensemble Offspring.
Paul Smith	2016	*Holding* Masks, for piano and toy piano	For the performance *Multiple, Keyboards*, 8 December, 2016, Theme and Variations Piano Showroom, Willoughby, Sydney.
Paul Smith	2016	*Clutter*, for two pianos	For the performance *Multiple, Keyboards*, 8^th^ December, 2016, Theme and Variations Piano Showroom, Willoughby Road, Sydney.
Clare Maclean	2016	*In the sea paths*, for viola and piano	For *Australia East and West – new music for viola and piano*, 26 July, 2016, Royal Conservatoire of Scotland, Glasgow.
Sean Botha		*Blood*, electronic instruments	CAPOW! Composers and Performers Out West, Western Sydney University, 20 October 2016.
Michael Hannan	2016	*Impromobile*, for piano	For the performance *Multiple, Keyboards*, 8 December, 2016, Theme and Variations Piano Showroom, Willoughby Road, Sydney.
Larry Sitsky	2015	*Letter from the* trenches, for mezzo, viola, percussion and piano	For the performance *War Letters*, Halcyon concert program, Ku-ring-gai Town Hall, 7 November 2015.
Andrián Pertout,	2016	*Musica Battuta* – double toy piano etude for one soloist, no. 438	For the performance *Multiple, Keyboards*, 8 December, 2016, Theme and Variations Piano Showroom, Willoughby Road, Sydney.
Nicole Murphy	2013	*Surface*, for viola and piano	For *Australia East and West – new music for viola and piano*, February, 14, 2013, Henry Jones Art Hotel, Hobart, Tasmania
**Program Notes Written By Professional Program Note Authors**			
Damien Ricketson	2012	*Hi Hat and Me* (2010) for percussion and voice, composer Matthew Shlomowitz	Ensemble Offspring, concert program, Campbelltown Arts Centre, 22 September 2012
Damien Ricketson	2012	*Cycles and Circles* (2012) for bass clarinet and percussion, composer James Humberstone	Ensemble Offspring, concert program, Campbelltown Arts Centre, 22 September 2012
Michael Hooper	2014	*A Tree Telling of Orpheus* (1990) for mezzo, soprano and chamber ensemble, composer David Lumsdaine	Halcyon concert program, St Bedes Anglican Church, Drummoyne, 12 July 2014
Elliott Gyger	2014	*Orphei Mysteria* (2008) for mezzo, soprano and chamber ensemble, composer Nigel Butterley	Halcyon concert program, St Bedes Anglican Church, Drummoyne, 12 July 2014
Jenny Duck-Chong	2016	*Woman’s Song, Midnight and Winter Kestrel* (1950/62) for mezzo, soprano and piano trio, composer Margaret Sutherland	Halcyon concert program, St Bedes Anglican Church, Drummoyne, 10 September 2016
Daniel Herscovitch	2016	*Piano Trio* (1991), composer Roger Smalley	Halcyon concert program, St Bedes Anglican Church, Drummoyne, 10 September 2016
Timothy Munro	2016a	*Nowhere and Forever* (2016) for viola and electronics, composer Natasha Anderson	Sydney Symphony Orchestra concert program, Carriageworks Sydney, 20 November 2016
Timothy Munro	2016b	*We Never Come Here* (2016) for chamber orchestra and electronics, composer: Alexander Garsden	Sydney Symphony Orchestra concert program, Carriageworks Sydney, 20 November 2016
Rachel Campbell	2008	*Beauty Boxes* (2008) for two percussionists, composer Rosemary Joy	Ensemble Offspring concert program, Carriageworks, Sydney, 10 December 2008
Rachel Campbell	2008	Swell (2008) for chamber ensemble, composer Michael Smetanin	Ensemble Offspring concert program, Carriageworks, Sydney, 10 December 2008
**Program Notes Written By Students**			
The 10 student authors wrote program notes about works they had composed or about works composed by other students. Program note authors are identified as *Student A* through to *J* in the text; composers, where referred to in extracts from the notes, are given pseudonyms.			
Student A Writing about the work of another student	1997	Prepared Piano work (1997)	University *Concert Practice* student program, 1997
Student B Writing about the work of another student	2015	Brass quintet (2015)	University concert program, 2015
Student C Writing about own composition	2015	Woodwind quintet (2015)	Youth Orchestra concert program, held at University, 2015
Student D Writing about own composition	2015	Work for piano, guitar and percussion ensemble (2015)	Youth Orchestra concert program, held at University, 2015
Student E Writing about own composition	2015	Work for brass quintet (2015)	Youth Orchestra concert program, held at University, 2015
Student F	2016	Guitar work (2016)	University concert program, 2016
Student G Writing about own composition	2016	Theremin work (2016)	University, student concert program, 2016
Student H Writing about own composition	1997	Work for Clarinet and Piano (1997)	University *Concert Practice* student program, 1997
Student I Writing about the work of another student	1997	Vocal work (1997)	University *Concert Practice* student program, 1997
Student J Writing about own composition	2015	Work for woodwind quintet (2015)	Youth Orchestra concert program, held at University, 2015
